# Enhanced Loran System Demodulation for Complex Receive Environments: A Novel Matched Correlation Method Integrating Notch Filtering and Pattern Modulation

**DOI:** 10.3390/s26020666

**Published:** 2026-01-19

**Authors:** Xiangyi Wang, Baorong Yan, Tao Jin, Yun Li, Shifeng Li, Shiyao Liu

**Affiliations:** 1 National Time Service Center, Chinese Academy of Sciences, Xi’an 710600, China; wangxiangyi24@mails.ucas.ac.cn (X.W.); jintao@ntsc.ac.cn (T.J.); liyun@ntsc.ac.cn (Y.L.); lishifeng@ntsc.ac.cn (S.L.); 2 University of Chinese Academy of Sciences, Beijing 100039, China; 3 Academic of Data Science, Xi’an Eurasia University, Xi’an 710065, China; liushiyao@eurasia.edu

**Keywords:** eLoran, demodulation, sky-wave interference, continuous wave interference

## Abstract

**Highlights:**

**What are the main findings?**

- A Matched Correlation method integrating Notch Filtering (MC-NF) is proposed to effectively suppress random noise and in-band continuous wave interference.
- The Pattern Modulation combined version (PMC-NF) further improves demodulation performance by approximately 2.8 dB compared to MC-NF.

**What are the implications of the main findings?**

- The proposed algorithms provide superior robustness and accuracy for eLoran systems operating in complex, high-interference environments.

**Abstract:**

Demodulation is a key technology for the enhanced Loran (eLoran) system to achieve positioning and timing, and it affects the final performance of the system. Based on the traditional matched correlation algorithm, this paper proposes a new matched correlation demodulation method with notch processing. Furthermore, by combining it with the pattern modulation of the eLoran system, the matched correlation integrate notch demodulation method is further modified to improve demodulation performance. Firstly, the data link of the eLoran system is introduced in detail, including the encoding and modulation processes, the influencing factors of received signals, and the evaluation methods in the demodulation process. Secondly, on the basis of the principle of the matched correlation (MC) demodulation algorithm, a matched correlation demodulation algorithm integrating notch processing (MC-NF) and a demodulation correlation algorithm combined with modulation patterns (PMC-NF) are proposed. And, an analysis of the key factors affecting demodulation performance is given. Next, the demodulation performance of the mentioned algorithms under the conditions of random noise, skywave, and in-band continuous wave interference is calculated in detail. A large number of experimental results show that notch processing performs excellently in suppressing random noise and in-band continuous wave interference, and it can greatly improve the demodulation performance of the traditional matched correlation algorithm. Moreover, PMC-NF is superior to MC-NF; approximately 2.8 dB at decoding the critical point.

## 1. Introduction

Positioning, Navigation, and Timing (PNT) systems are critical infrastructure for modern nations and societies, with their importance permeating various aspects including the national economy, defense security, and public services. From precision agriculture and intelligent transportation to the synchronized operation of power grids, and from precise timestamps for mobile communications and financial transactions to the precise command of military operations, highly reliable and accurate PNT services have become a crucial cornerstone supporting the efficient functioning of society and safeguarding national strategic security [1,2,3,4,5]. Global Navigation Satellite Systems (GNSSs), particularly the development and application of systems like GPS and BeiDou, have brought revolutionary changes to PNT. They have significantly enhanced the global coverage, civilian accessibility, and technological maturity of PNT services, transforming once expensive and complex capabilities for precise positioning and timing into a public service accessible to all at low cost or even for free [6,7]. Despite the revolutionary convenience offered by GNSSs, they possess inherent weaknesses and vulnerabilities, such as signals being easily blocked or interfered with and limited anti-jamming and anti-spoofing capabilities. These shortcomings prevent GNSSs from solely providing reliable PNT services [8,9,10,11,12]. The enhanced Loran (eLoran) system transmits high-power, low-frequency ground-wave signals that are not easily blocked, interfered with, or spoofed. It boasts strong anti-jamming and anti-spoofing capabilities, high reliability, wide coverage, and possesses advantages such as differential enhancement capability. These attributes allow it to compensate for many of GNSSs’ deficiencies, serving as a reliable backup to ensure service continuity during GNSS outages or denial, providing users with credible backup PNT information, and enhancing overall PNT service assurance [13,14,15,16]. In recent years, many countries worldwide have embarked on resuming the research, development, and construction of eLoran systems [17,18]. The construction of the eLoran system represents a crucial strategic investment for ensuring national economic security, defense security, and public security. It is a key pillar in transitioning the national PNT architecture from over-reliance on GNSS to multi-source integration, and it is an indispensable component in building a national integrated space–air–ground PNT system [19,20,21].

The eLoran receiver is the end device that enables the timing and positioning functions of the eLoran system, playing a pivotal role within the entire system [22]. Signal reception and processing in eLoran is the core process of converting terrestrial low-frequency signals into usable timing, navigation, and positioning information. Demodulation and decoding form the most fundamental yet critical step in this processing chain. They are responsible for extracting useful data containing key information such as time and position from the received low-frequency signals, directly impacting the timing accuracy and data transmission reliability of the eLoran system. Recent years have witnessed extensive research on eLoran demodulation techniques by scholars worldwide. Currently, the predominant method employed in eLoran systems is envelope phase detection (EPD) [23,24]. This technique derives phase differences by sampling the envelopes of two orthogonal signals, thereby converting the phase shifts of 0° or ±36° induced by eLoran PPM modulation into corresponding modulation codewords for demodulation. However, EPD demonstrates high vulnerability to in-band continuous wave interference (CWI) and noise, revealing considerable limitations in both anti-interference and noise resistance capabilities. In 2007, Lo (2007) [25] introduced the signal matching correlation–pulse position detection (SMC-PPD) technique, which relies on time-domain signal sliding correlation. Nevertheless, with increasing interference and noise levels, the frequency of errors in peak position identification rises substantially, resulting in elevated bit error rates. In 2020, Yuan (2020) [26] developed the envelop correlation-phase detection (EC-PD) algorithm, integrating two envelope correlation approaches tailored for different scenarios: moving average-cross correlation (MA-CC) and matched correlation (MC). Their investigation into skywave effects on signal-to-noise ratio (SNR) gain enabled adaptive switching, thereby enhancing demodulation accuracy and anti-interference performance in complex electromagnetic environments, although it remains fundamentally a phase detection methodology. More recently, in 2022, Lyu (2022) [27] proposed a log-likelihood ratio (LLR)-based algorithm that computes and compares the probability of each transmitted information bit being ‘0’ or ‘1’, bypassing conventional demodulation to directly decode signals. While this approach improved decoding success rates to some extent, it failed to account for the influence of diverse interference conditions on probability calculations, necessitating further derivation and validation. In 2024, Liu (2024) [28] proposed a multi-class support vector machine (MSVM)-based eLoran demodulation algorithm. This method utilizes machine learning technology, constructing multi-dimensional feature vectors and training a multi-class SVM model to classify and demodulate pulse position modulation signals. Experiments showed its significant superiority over traditional methods in anti-noise and anti-interference performance, but the algorithm’s complexity poses challenges for receiver hardware implementation. In the same year, Liu (2024) [29] further proposed the phase spectrum smoothing demodulation (PSSD) algorithm. Through techniques like windowed sampling, phase spectrum smoothing, and weighted phase difference calculation, it effectively suppresses in-band noise and continuous wave interference, demonstrating superior and stable demodulation performance under different SNR and interference environments. However, its application on actual hardware platforms requires further optimization and verification.

Following the previous discussion, to further advance the demodulation capabilities of eLoran systems under complex electromagnetic conditions, this paper proposes an enhanced data demodulation method based on notch processing. Building upon the traditional matched correlation algorithm, this method innovatively integrates the notch filtering method and the system’s inherent pattern modulation to form an anti-interference enhanced demodulation scheme. The primary theoretical contributions of this work can be summarized in the following key aspects:

Firstly, a matched correlation demodulation algorithm integrating notch processing (MC-NF) is proposed. This method suppresses the impact of random noise through notch filtering of the fixed-frequency eLoran signal; meanwhile, the identification and notch processing of in-band CWI reduce the influence of interference, effectively enhancing the demodulation performance of the traditional matched correlation algorithm.

Secondly, a demodulation correlation algorithm combined with modulation patterns (PMC-NF) is proposed. Building upon MC-NF, this method further improves demodulation performance by increasing the data length, achieving a significant leap in performance. This greatly enhances the system’s reliability and service quality, holding significant practical importance for consolidating the role of the eLoran system as an effective supplement and backup to GNSS.

Finally, this study validates the effectiveness of the MC-NF and PMC-NF methods through extensive simulation experiments, providing a new technical pathway for eLoran demodulation in complex receive environments and outlining the direction for subsequent application verification in actual receivers and algorithm optimization.

## 2. Materials and Methods

### 2.1. Background Principles

This section mainly introduces the signal modulation process of eLoran, specifically compares the differences between the signal received and the ideal signal at the transmitter, and determines the reference pulse for demodulation processing at the receiver. Simultaneously, it presents the calculation method and evaluation principles for demodulation performance.

#### 2.1.1. Signal Modulation of ELoran

**Figure 1:**
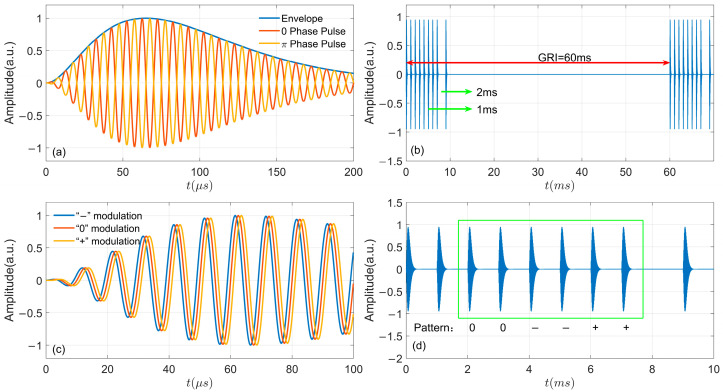
Modulation of eLoran: (**a**) Envelope and standard pulse; (**b**) Master station pulse group with a GRI = 60 ms; (**c**) Three-state pulse position modulation; (**d**) Modulation pattern.

As specified by the International Telecommunication Union (ITU), the operational frequency band for eLoran systems primarily spans 90 to 110 kHz, utilizing a carrier center frequency of 100 kHz. The standardized, unmodulated Loran-C pulse waveform is mathematically defined by Formula (1) [30,31]:

(1) $$s\left(t\right)=\left\{\begin{array}{c} A{\left(t-\tau \right)}^{2}\exp\left(-2\frac{\left(t-\tau \right)}{65}\right)\sin\left(2\pi f_{c}t+P_{c}\right),\;\;\tau \ll t\ll 65+\tau \;\\ \;\;\;\;\;\;\;\;\;\;\;\;\;0,\;\;\;\;\;\;\;\;\;\;\;\;\;\;\;\;\;\;\;\;\;t<\tau \end{array}\right.$$

where *A* is a normalization factor determining the peak amplitude; *t* denotes time in μs; *τ* represents the envelope-to-cycle difference in µs; $f_{c}$ = 100 kHz is the carrier frequency; and $P_{c}$ = 0 or π indicates the phase code parameter in radians. The standard waveform incorporating phase encoding is shown in Figure 1a.

ELoran systems employ a pulse group format for signal transmission, with data communication achieved by modulating the phase of individual pulses. Within a given group repetition interval (GRI), the master station transmits a sequence of nine pulses per group. The first eight pulses are spaced at 1-millisecond intervals, followed by a 2-millisecond interval before the ninth pulse. In contrast, each secondary station transmits only the initial eight pulses. To support station identification and precise timing, each transmitting chain is assigned a unique GRI [32,33]. Figure 1b gives a sample of a group with GRI = 60 ms.

The eLoran system is equipped with Eurofix data link technology, which enables the modulation of communication data directly onto the transmitted pulse groups [34,35]. The eLoran system employs a three-state pulse position modulation (PPM) with a 1 μs modulation interval on the 3rd~8th pulses of each pulse group. This modulation method generates three signal patterns: (1) if the transmission phase is advanced by 1 μs, it represents the symbol “−”; (2) if the transmission phase has no displacement, it represents the symbol “0”; (3) if the transmission phase is delayed by 1 μs, it represents the symbol “+”. These patterns are illustrated in Figure 1c and correspond to the characters “−”, “0”, and “+”, respectively [36,37]. One pulse group corresponds to one pattern, and the receiver decodes the target information by identifying the pattern, shown in Figure 1d. There are 141 types of balanced modulations among the total of 3^6^ = 729 possible modulation combinations for the 3rd~8th pulses. Among them, a set of 128 different balanced combinations is utilized in eLoran, establishing a direct mapping to the conventional 128-character ASCII code common in computer systems [23,38]. The specific corresponding relationships are shown in Table 1. It should be noted that every 30 patterns in the eLoran data link form one data frame, which is equivalent to 210 bits of binary data. Among them, the data information contains 56 bits [39]. Furthermore, the data frame incorporates 14 bits of Cyclical Redundancy Check (CRC) code and 140 bits of Reed–Solomon (RS) forward error-correcting code, employed for detecting and correcting demodulation errors to enhance data integrity [40,41,42].

#### 2.1.2. Received Signal

**Figure 2:**
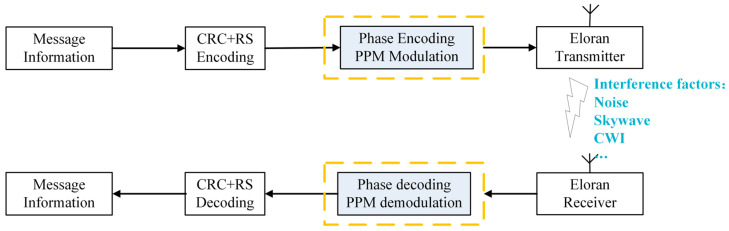
Processing flow of the eLoran data link and interference factors of received signal.

In this section, the differences between the signals actually received by the eLoran receiver and the ideal simulated signals are introduced. Due to the influence of the real propagation environment, the received signal inevitably contains interfering components such as random noise, continuous wave interference (CWI), and skywave, which cause distortion of the eLoran signal during propagation. The random noise interferes with the amplitude and phase of the true useful signal, masking the true structure of the signal; The CWI, especially the CWI in the specific frequency band, causes concentrated interference to the signal, leading to local distortion of the frequency spectrum; the existence of skywaves causes multipath propagation of the signal, resulting in signal superposition and waveform distortion. In addition, the system is also affected by factors such as Envelope-to-Cycle Difference (ECD), which further causes non-ideal changes in the pulse envelope shape [43,44,45]. Figure 2 gives the processing flow of the eLoran data link and interference factors of received signal, highlighting the distortion of the received signal during propagation. Therefore, this paper mainly focuses on the research of data demodulation issues in the eLoran system under interference conditions, with emphasis on addressing the impact of typical interference types (including random noise, skywave interference, and CWI) on demodulation performance. The ultimate objective is to optimize the demodulation performance and reliability of eLoran receivers when operating in challenging, non-ideal environments.

**Figure 3:**
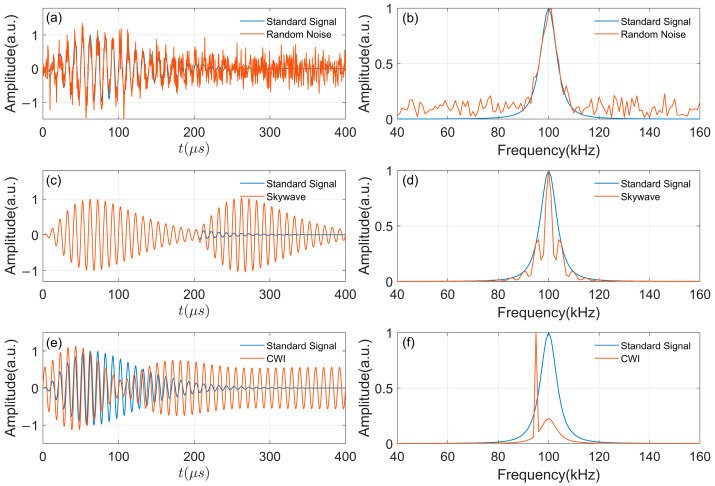
ELoran wingle-pulse signal with different types of interference: (**a**) Time-domain comparison of noisy signals; (**b**) Frequency-domain comparison of noisy signals; (**c**) Time-domain comparison of skywave interference; (**d**) Frequency-domain comparison of skywave interference; (**e**) Time-domain comparison of CWI; (**f**) Frequency-domain comparison of CWI.

As mentioned earlier, when the received signal contains various interferences, corresponding changes in the received signal will occur in both the time domain and frequency domain. Figure 3 shows the changes in the time domain and frequency domain of the signal when the received signal contains random noise, sky-wave, and in-band continuous wave interference. Figure 3a and Figure 3b show the effects of random noise on the time domain and frequency domain of the signal, respectively, with SNR = 5 dB. It can be seen from the figures that random noise destroys the smoothness of the time-domain signal and changes the amplitude of signals at all frequencies. Figure 3c,d show the influences of skywave interference, and Figure 3e,f show the influences of CWI.

#### 2.1.3. Evaluation of Demodulation Performance

**Figure 4:**
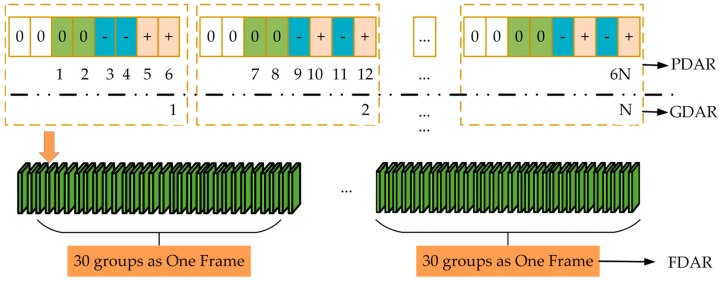
The different demodulation accuracy rate of the receiving system.

In research on the key technologies of the eLoran system, the Demodulation Accuracy Rate (DAR) of the receiving system is a critical indicator for measuring the capability of receiving equipment. As shown in Figure 4, DAR can be divided into Pulse Demodulation Accuracy Rate (PDAR), Group Demodulation Accuracy Rate (GDAR), and Frame Demodulation Accuracy Rate (FDAR) according to different evaluation granularities. This multi-granularity accuracy evaluation mechanism helps to analyze the demodulation performance of the system under different interference conditions more comprehensively and objectively.

PDAR is the accuracy rate calculated with a single pulse as the basic unit. If the total number of pulses is 6N and the number of correctly demodulated pulses is denoted as $P_{right}$, its calculation formula is:

(2) $$PDAR=\frac{P_{right}}{6N}\times 100\%$$

where 0≤PDAR≤100%.

GDAR is the accuracy rate calculated with a pulse group (composed of eight single pulses) as the unit. It is defined as the ratio of the number of correctly demodulated pulse groups (denoted as $P_{group}$) to the total number of groups (*N*). A pulse group can only be determined as correct if all pulses in the group are demodulated correctly, with the first two pulses excluded from the demodulation calculation. This avoids the impact of correct demodulation of individual pulses within the group on the overall demodulation performance. Considering that the demodulation of each single pulse in the group is an independent event, its calculation formula can be expressed as follows:

(3) $$GDAR=\frac{P_{group}}{N}\times 100\%={\left(\frac{P_{right}}{6N}\right)}^{6}\times 100\%$$

According to the above formula, GDAR ≤ PDAR, which means that under the same conditions, the accuracy rate represented by GDAR is lower than that represented by PDAR.

Similarly, FDAR uses an entire frame of information composed of 30 pulse groups as the judgment unit with the formula given in (4). It is defined as the ratio of the number of correctly demodulated data frames after RS decoding and CRC verification (denoted as $P_{frame}$) to the total number of frames ($\frac{N}{30}$). By combining RS (30, 10) decoding and CRC verification, the value of GDAR can be linked to the value of FDAR. Among the 30 pulse groups, if the number of incorrect pulse groups is 10 or less, i.e., GDAR>66.7%, the entire frame of data can be correctly demodulated using RS correction. If the number of incorrect pulse groups exceeds 10, the demodulation of the data frame will not be achievable. *GDAR* = 66.7% is a key parameter for judging demodulation performance, which will be marked clearly in the subsequent discussion.

(4) $$FDAR=\frac{P_{frame}}{\frac{N}{30}}\times 100\%$$

### 2.2. Method Description

This section will introduce commonly used matched correlation algorithms, then it will present a new algorithm called Matched Correlation Integrating Notch Filtering (MC-NF). Furthermore, Pattern Matched Correlation Integrating Notch Filtering (PMC-NF) for eLoran signals demodulating is derived. We will provide a thorough description and comparison of the processing logic, algorithmic details, and the advantages and disadvantages of each method.

#### 2.2.1. Matched Correlation

**Figure 5:**
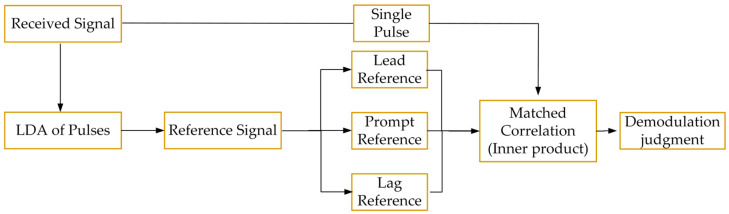
Matched correlation demodulation technology block diagram.

Matched Correlation (MC) is one of the most common methods in digital signal processing [26,27]. It measures the similarity degree between two signals and is often used for target signal identification and synchronization. Considering the modulation characteristics of eLoran and the "distortion" of the received signal, an unmodulated pulse is selected as the reference signal. Three templates are constructed in the form of PPM, corresponding to “−”, “0”, and “+” modulations, respectively. The received signal is correlated (inner Product) with each of these templates separately; the maximum correlation result indicates the highest similarity between the received signal and that specific template, and this is used to achieve pulse demodulation. The technical block diagram of the scheme is shown in Figure 5. It should be noted that the reference signal in Figure 5 is obtained through linear digital averaging (LDA), which will be described and analyzed specifically in the following section.

The matched correlation algorithm uses inner product results for demodulation and exhibits strong anti-noise performance. However, in the mentioned processing flow, the received signal is directly correlated with the three templates without prior processing, which limits its demodulation performance to a certain extent. Meanwhile, if the signal contains CWI, especially when the number of continuous wave cycles within the single-pulse duration (1 ms) is not an integer, the demodulation performance of this method will be significantly reduced.

#### 2.2.2. Matched Correlation Integrating Notch Filtering

**Figure 6:**
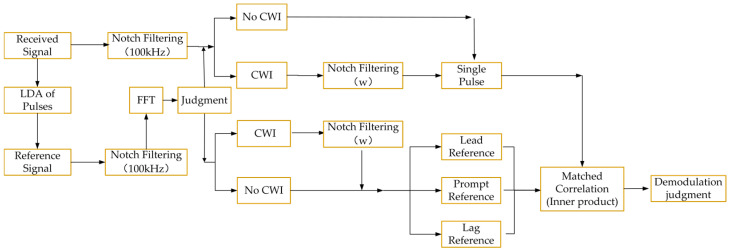
Matched correlation integrating notch filtering demodulation block diagram.

In Section 3.1, the deficiencies existing in the MC algorithm are presented. To address these issues that affect demodulation performance, this section combines notch filtering with the MC method and presents the processing flow of Matched Correlation Integrating Notch Filtering (MC-NF), as shown in Figure 6. In the MC-NF method, both the reference pulse and the pulse to be demodulated undergo notch filtering. Meanwhile, it adds the judgment of continuous wave interference in the signal and performs notch filtering on the interference in subsequent processing. The two notch filtering progresses suppress random noise and in-band continuous wave interference, respectively, thereby improving demodulation performance. A detailed evaluation is provided in the subsequent simulation calculations.

To clearly articulate the internal logic, the execution procedure is performed in the following distinct steps:

Step 1: Reference Signal Acquisition. A high-SNR reference pulse, denoted as $s_{ref}(t)$, is obtained by applying LDA to the received signal over *N* groups. This accumulated reference serves as the baseline for subsequent correlation processing.

Step 2: Interference Detection and Notch Filtering. This step mitigates interference prior to correlation. The system computes the FFT of the received signal. If the normalized amplitude difference between the peak interference frequency and the carrier frequency exceeds the threshold of 0.15, the algorithm identifies the CWI. Subsequently, an IIR notch filter (Q=5) is designed at the interference frequency. Crucially, this notch filtering process is concurrently applied to both the received signal x(t) and the reference signal $s_{ref}(t)$ to ensure signal consistency and maximize interference suppression.

Step 3: Matched Correlation and Decision. The filtered reference signal is then used to construct local templates corresponding to the PPM symbols. The method calculates the cross-correlation between the filtered received signal and these templates, selecting the symbol associated with the maximum correlation peak.

#### 2.2.3. Pattern Matched Correlation Integrating Notch Filtering

**Figure 7:**
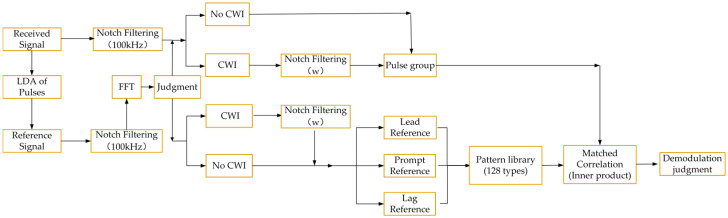
Pattern Matched Correlation Integrating Notch Filtering demodulation block diagram.

This section will introduce the modulation pattern of eLoran into the MC-NF method, whose form has been mentioned in the background introduction. For convenience, the demodulation form that combines MC-NF with the pattern is described as Pattern Matched Correlation Integrating Notch Filtering (PMC-NF), shown in Figure 7. Procedurally, the PMC-NF method inherits the Interference Detection and Notch Filtering logic established in the MC-NF method. Compared with the MC-NF method, the reference pulse is no longer a set of single PPM pulses. Instead, the algorithm utilizes a Pattern Library containing all 128 valid modulation combinations. Group-level templates are constructed by assembling the filtered reference pulses according to the delays of each pattern. Similarly, the signal to be demodulated should contain the data of a pulse group. The greatest advantage of this method is that it avoids unbalanced modulation constructed during single-pulse demodulation and improves demodulation performance. A detailed evaluation is provided in the subsequent simulation calculations.

#### 2.2.4. Key Modules Analysis

##### Sampling Rate

The three standard PPM pulses of eLoran without noise are stated as $\;s_{k}\left(t\right)$, where $k\in \left\{0,+,-\right\}$. At the same time, the received noisy signal of eLoran is expressed as follows:

(5) $$x\left(t\right)=s_{k}\left(t\right)+n\left(t\right)$$

Here, $x\left(t\right)$ is a single pulse, and $n\left(t\right)$ denotes the noise at this moment, which is zero-mean Gaussian white noise with a power spectral density of $\sigma^{2}$. If the sampling rate is $f_{s}$, then the sampling interval is $∆t=\frac{1}{f_{s}}$. A discrete signal can be expressed as:

(6) $$S_{k}=s_{k}\left(i∆t\right)+n\left(i∆t\right)$$

where i=0,1,2,…,N−1 denotes the number of samples. The result of the matched correlation (inner product) can be expressed as follows:

(7) $$Z_{k}=〈S_{m},S_{k}〉=\sum_{i=0}^{N-1}s_{m}\left[i\right]\cdot \left(s_{k}\left[i\right]+n\left[i\right]\right)\;\;=\sum_{i=0}^{N-1}s_{m}\left[i\right]\cdot s_{k}\left[i\right]+\sum_{i=0}^{N-1}s_{m}\left[i\right]\cdot n\left[i\right]$$

In order to analyze the impact of the sampling rate on demodulation performance, the correlation results are divided into two parts for discussion.

(1) Signal power item:

(8) $$\sum_{i=0}^{N-1}s_{m}\left[i\right]\cdot s_{k}\left[i\right]\approx f_{s}\int_{0}^{T}s_{k}\left(t\right)s_{m}\left(t\right)dt=f_{s}\cdot R_{km}$$

where T=N∆t is the total sampling time, and $R_{km}$ is the integral result of cross-correlation. It is in a matched state when k=m, and the signal energy reaches its maximum when $R_{kk}=E_{s}$. Similarly, it is in an unmatched state when k≠m, $R_{km}=E_{c}$ with $E_{c}<E_{s}$. It can be seen that the signal energy term is proportional to the sampling rate $f_{s}$.

(2) Noise Interference Term:

(9) $$N_{k}=\sum_{i=0}^{N-1}s_{m}\left[i\right]\cdot n\left[i\right]$$

Since $n\left[i\right]$ is zero-mean Gaussian white noise with $E\left(n\left[i\right]\right)=0$ and $s_{m}\left[i\right]$ is a deterministic useful signal independent of noise, the mathematical expectation of the noise interference term is zero:

(10) $$E\left[\sum_{i=0}^{N-1}s_{m}\left[i\right]\cdot n\left[i\right]\right]=0$$

And the variance of the noise interference term is derived as follows:

(11) $$Var\left(N_{k}\right)=\sum_{i=0}^{N-1}{s_{m}\left[i\right]}^{2}\cdot Var\left(n\left[i\right]\right)=\sigma^{2}f_{s}\int_{0}^{T}{s_{m}\left(t\right)}^{2}dt=\sigma^{2}f_{s}E_{s}$$

It can be seen that the variance in the noise interference term is also proportional to the sampling rate $f_{s}$.

The analysis of the correlation result components shows that the useful signal power term and the variance of the noise interference term are related to both the sampling rate and the noise power spectral density. The difference in the matched state and unmatched state is stated as follows:


(12) $$Z_{k}-Z_{m}=f_{s}\left(E_{s}-E_{c}\right)+\left(N_{m}-N_{k}\right)$$

Polarity decision: In the matched correlation demodulation, the probability of a correct decision is expressed in Formula (13), where $\Phi \left(\cdot \right)$ denotes the cumulative distribution function (CDF) of the standard normal distribution. This confirms that increasing the sampling rate helps accumulate more effective signal information, thereby improving demodulation accuracy under the same SNR.


(13) $$\begin{array}{c} P_{correct}=P\left[f_{s}\left(E_{s}-E_{c}\right)+\left(N_{m}-N_{k}\right)>0\right]\\ =\Phi \left(\frac{f_{s}\left(E_{s}-E_{c}\right)}{\sqrt{\sigma^{2}f_{s}E_{s}}}\right)=\Phi \left(\sqrt{f_{s}}\cdot \sqrt{\frac{{\left(E_{s}-E_{c}\right)}^{2}}{\sigma^{2}E_{s}}}\right) \end{array}$$

Table 2 gives the result of the matched correlations for different sampling. As the sampling rate increases, the difference gradually becomes proportional to the sampling rate.

**Figure 8:**
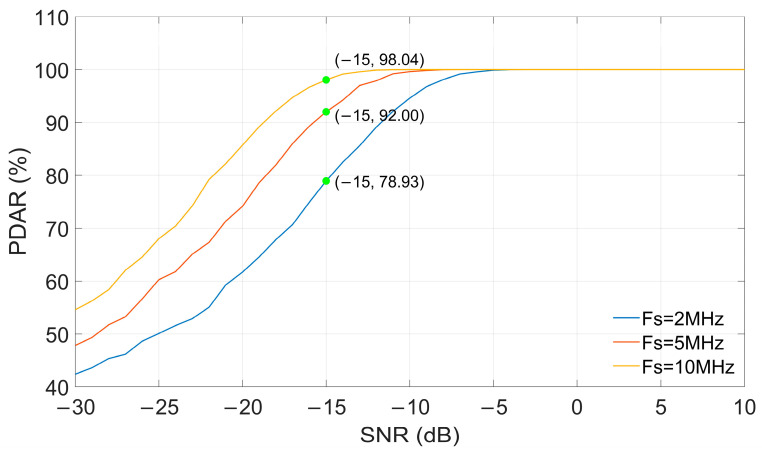
The influence of sampling rate on PDAR.

Figure 8 shows the relationship between the PDAR of the MC algorithm and the SNR of the signal under different sampling rates. Here, the reference pulse is a noise-free standard signal, and the received signal contains only random noise. It can be seen from the figure that when the SNR is constant, the value of FDAR increases as the sampling rate increases. When the SNR is −15 dB, the FDAR values are 98.04%, 92.00%, and 78.93%, respectively, corresponding to sampling rates of 10 MHz, 5 MHz, and 2 MHz.

##### Reference Signal Preprocessing

**Figure 9:**
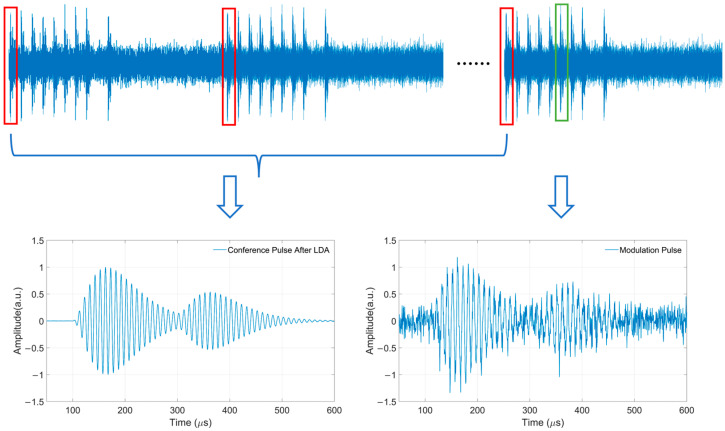
The reference signal pulse and the pulse to be demodulated.

In the analysis of the sampling rate, the power spectral density of noise has a certain impact on demodulation performance, including both the reference signal and the signal to be demodulated. Considering the modulation characteristics of the eLoran signal, the first two pulses in a pulse group are not subjected to PPM. These two pulses can be processed with LDA to suppress the noise. Figure 9 gives the difference between the reference signal after LDA and the pulse for demodulation. As above, the first two unmodulated pulses in the eLoran pulse group are the same, shown as follows:

(14) $$x\left(t\right)=s_{0}\left(t\right)+n\left(t\right)$$

And the Signal-to-Noise Ratio (SNR) of signal pulse is described as follows:

(15) $${SNR}_{in}=10lg\left(\frac{P_{s0}}{\sigma^{2}}\right)$$

where $P_{s0}$ is the average power of the useful signal, and $\sigma^{2}$ is the average power of the noise. After LDA processing of M signals,

(16) $$y\left(t\right)=\frac{1}{M}\sum_{i=1}^{M}x_{i}\left(t\right)=s\left(t\right)+\frac{1}{M}\sum_{i=1}^{M}n_{i}\left(t\right)$$

and the SNR is improved:

(17) $${SNR}_{out}=10lg\left(P_{s0}/{(\sigma }^{2}/M)\right)=10\mathrm{lg}\left(M\right)+{SNR}_{in}$$

That is, the SNR of the reference signal after LDA increases with the logarithm of M. If M = 10, the SNR is improved by 10 dB; when M = 100, the SNR is improved by 20 dB. While the SNR of the received signal is relatively low, more pulses need to participate in the LDA to achieve the required SNR of the reference pulse. However, as the number of superpositions increases, the SNR gain is no longer so significant. The impact of the reference signal SNR on demodulation performance will be discussed in the following section.

##### Notch Filtering Design

Notch filtering is a common digital filtering method that can achieve strong attenuation of signals at specific frequencies, filtering out certain interference signals [46]. The application condition of a notch filter is that the frequency of the signal to be notched is known. Compared with Least Mean Square filtering or Wiener filtering, there is no need to introduce a desired signal into the system as a reference signal. Due to its excellent notch performance, notch filters have been widely used in the field of signal processing, including useful signal extraction.

The Z-domain transfer function of a notch filter is expressed as follows:

(18) $$H\left(Z\right)=\frac{1-2\cos\left(\omega_{0}\right)Z^{-1}+Z^{-2}}{1-\frac{2\cos\left(\omega_{0}\right)}{\left(1+\alpha \right)Z^{-1}}+\frac{1-\alpha }{\left(1+\alpha \right)Z^{-2}}}$$

**Figure 10:**
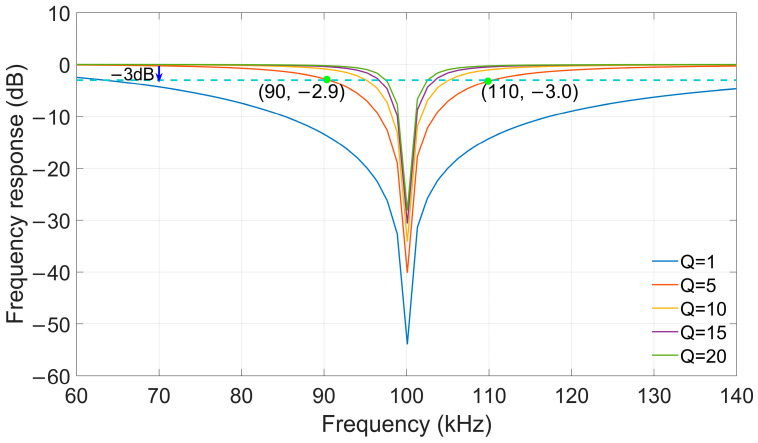
The frequency responses of the notch filter with different Q values.

Here, $\omega_{0}$ denotes the angular frequency of the signal to be notched, and α is calculated as $\alpha =\frac{\sin\left(\omega_{0}\right)}{2Q}$. The parameter Q represents the quality factor, which characterizes the frequency selectivity of the notch filter. Specifically, a larger Q value corresponds to a narrower notch bandwidth. The filter parameters are set based on the frequency response characteristics of the notch filter and the frequency-domain features of the eLoran signal. The energy of the eLoran signal is concentrated in the frequency range of 90 kHz to 110 kHz, which corresponds to a bandwidth (*BW*) of 20 kHz and a carrier frequency ($f_{0}$) of 100 kHz. To match the 20 kHz bandwidth requirement of the eLoran signal, the quality factor is determined by the ratio of the center frequency to the bandwidth ($Q=\frac{f_{0}}{BW}$). Consequently, with $f_{0}=100\;kHz$ and BW=20 kHz, the Q value is set to 5 in this study. Figure 10 shows the frequency responses for different Q values and the noisy eLoran signal.

#### Interference Decision

**Figure 11:**
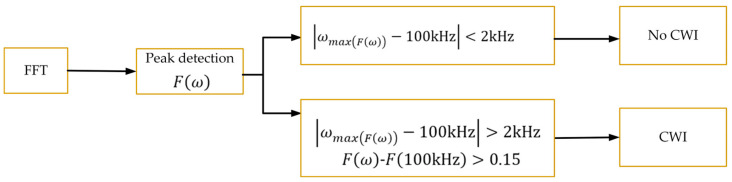
Block diagram of interference decision method.

In both processing flows described in Section 3.2 and Section 3.3, it is necessary to make a judgment on the reference signal to determine whether interference exists in the received signal. Among these interferences, the CWI in-band has a relatively obvious impact and can be analyzed in the frequency domain. If the power of the interference signal is much smaller than that of the eLoran signal, the amplitude of the interference in the frequency domain is not obvious, and the frequency corresponding to max(*F*(*ω*)) is around 100 kHz and there is no CWI. On the contrary, the amplitude of the interference signal in the frequency domain is relatively large, which is different from 100 kHz. In this paper, the frequency-domain amplitude is normalized after FFT. If the difference between the amplitude of the interference signal and that of the eLoran signal is greater than 0.15, the signal is determined to contain CWI. The specific interference judgment and processing flow is shown in Figure 11.

## 3. Results

This section will compare the proposed MC-NF algorithm with the traditional MC algorithm using the index of PDAR. Furthermore, the modified MC-NF algorithm named PMC-NF algorithm will be discussed. Meanwhile, the demodulation performance is represented by GDAR, comparing the MC-NF algorithm and PMC-NF. All discussions include scenarios involving random noise, skywave, and in-band continuous wave interference.

### 3.1. Experiment Design

The experimental environment design is given below:

- Experiment environment: Windows 10 operating system, Intel Core i7 processor, 16 GB memory. Sometimes, to save computation time, it is necessary to ensure the capability of parallel computing.
- Input signal: Continuous modulated pulse group signals.
- Sampling rate: 2 MHz is chosen in the experiment.
- Notch filtering parameter: Considering the characteristics of the enhanced Loran signal, the parameter of the notch filtering is set to *Q* = 5.
- SNR: There are two types of SNRs that need to be considered. One is the SNR of the reference pulse, and the other is the SNR of the signal to be demodulated. Signal-to-noise ratios from −30 dB to 10 dB are chosen for the demodulated signal by adding Additive White Gaussian Noise (AWGN). To ensure experimental reproducibility, the noise generation is strictly defined: the noise amplitude is calculated based on the instantaneous signal amplitude at the standard cycle identification point (25 µs), denoted as $A_{25µs}$ (approx. 0.506 in the normalized model). The standard deviation of the added noise, $\sigma_{n}$, is determined by $\sigma_{n}=A_{25µs}\times 10^{-SNR/20}$. The step size is set to 1 dB in this paper. If the number of pulses selected to participate in LDA is 100, the minimum SNR of the reference pulse is −10 dB; this represents the case with the worst reference signal in this paper.
- Skywave: In this case, the time delay and the amplitude ratio of the skywave relative to the ground wave are two factors affecting demodulation performance. Since the skywave usually lags behind the ground wave by 37.5 µs, the range of the delay value is set to 40–300 µs, and the SIR range is selected between 0 dB and −6 dB.
- CWI in band: single-frequency interference is considered. Interference frequency and amplitude are two considered factors. In-band interference can be divided into two types based on signal frequency. One type occurs when the single eLoran pulse duration (1 ms) exactly contains an integer number of cycles of the interference signal; the other type occurs when the duration of a single eLoran pulse contains a non-integer number of cycles of the interference signal. In this paper, these two cases are both discussed at different amplitudes, depicted by SIRs. The SIR is selected at −6 dB and 12 dB.
- Evaluation index: This study utilizes the Demodulation Accuracy Ratio as the primary evaluation index, where PDAR and GDAR are used for comprehensive evaluation of MC-NF and PMC-NF, respectively.
- Monte Carlo Simulations: To ensure the statistical reliability and robustness of the experimental results, the number of Monte Carlo simulations is set to 10,000 for the validation of each algorithm. For MC-NF, the received signal for demodulation consists of 10,000 randomly modulated pulses. But for PMC-NF, the received signal for demodulation consists of 10,000 randomly modulated pulse groups.

### 3.2. Performance Evaluation of PDAR

In this section, the demodulation performance of the MC-NF algorithm is mainly calculated, including the impacts of random noise, skywave interference, and CWI in-band. Additionally, the MC algorithm without notch filter processing is also calculated, which is used for comparison with the results obtained with notch filter processing.

#### 3.2.1. Random Noise

**Figure 12:**
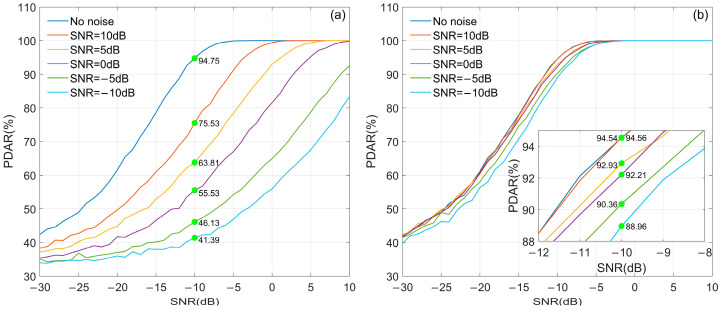
PDAR comparison of different SNR reference signals. (**a**) MC method, (**b**) MC-NF method.

Random noise is the most common interference factor in received signals, and this section mainly analyzes the impact of random noise on PDAR. Since the signal contains only random noise, no CWI is detected in the received signal after FFT transformation and interference judgement. Figure 12 shows the PDAR of MC and the MC-NF method under different SNRs of the reference pulse, respectively. The sampling rate is 2 MHz, and the number of simulations is 10,000 times under each certain reference pulse. It can be seen that the SNR of the reference pulse has a significant impact on the demodulation results: the higher the SNR of the reference pulse, the better the demodulation performance. Compared with the MC algorithm shown in Figure 12a, the MC-NF algorithm in Figure 12b is less dependent on the reference pulse. This is because both the received signal to be demodulated and the reference signal have undergone notch filtering, which suppresses noise and improves the SNR.

**Figure 13:**
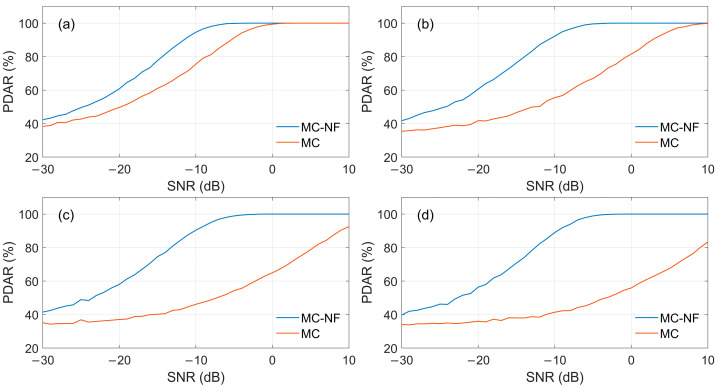
PDAR comparison of different SNR reference signals: (**a**) SNR = 10 dB; (**b**) SNR = 0 dB; (**c**) SNR = −5 dB; (**d**) SNR = −10 dB.

Figure 13 presents a comparison of PDAR values calculated by the MC-NF and MC algorithms, corresponding to different reference pulses. The main factor determining demodulation performance is random noise, especially the noise in the reference pulse. In Figure 13a–d, the reference pulses contain noise, with their SNRs corresponding to 10 dB, 0 dB, −5 dB, and −10 dB respectively. It can be seen from the figure that the demodulation performance of MC-NF is better than that of MC under all circumstances. When the SNR of the reference signal is relatively low, the difference becomes more pronounced. As shown in Figure 13d, when the SNR of the received signal is −10 dB, the demodulation accuracy of MC-NF is 88.96%, while that of MC is only 41.39%. The calculation results indicate that MC-NF is more suitable for demodulating signals under low SNR conditions.

#### 3.2.2. Skywave

This section discusses the performance of the demodulation and differences between the MC-NF and MC algorithm in the presence of skywave. In the received signal, the skywave signal’s frequency is identical to the ground wave signal’s frequency except for a certain time delay, making it difficult to separate the skywave signal from the composite signal. However, the time delay of the skywave relative to the ground wave will affect the demodulation performance. Additionally, the amplitude of the skywave is another factor. Therefore, the impacts of time delay and SIR on demodulation performance are considered in detail.

Simulation experiments show that when the received signal contains skywave components, under the same set of conditions, the demodulation performance of the MC-NF method is superior to that of the MC method, which are similar to those in the case of random noise.

1. Skywave Delay

**Figure 14:**
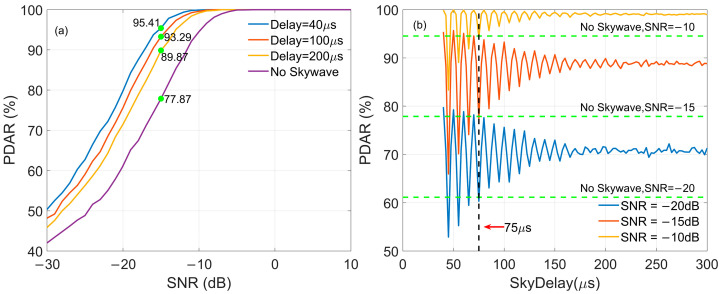
The impact of skywave delay on PDAR: (**a**) the PDAR for different delays; (**b**) the PDAR via delay for different SNRs.

Figure 14 shows the impact of skywave on PDAR, which depicts the demodulation performance of eLoran using MC-NF. Here, the reference pulse contains no random noise, and SIR = 0 indicates that the skywave and groundwave have the same signal amplitude. Figure 14a presents the PDAR value of the received signal as a function of SNR at different skywave delay value. It can be seen from the figure that when the skywave delay value is constant, the PDAR value increases as the SNR in the signal rises. This indicates that the demodulation performance improves as the SNR increases. Figure 14a also shows that the PDAR values are affected by the skywave delay values. The PDAR values are 95.41%, 93.29, and 89.87%, corresponding to delay values of 40 μs, 100 μs, and 200 μs, respectively, when the received signal’s SNR is −10 dB. These results are higher than the value of no skywave interference. Additionally, the PDAR values related to the skywave delay value are simulated shown in Figure 14b, where the composite pulses for demodulation with SNR −20 dB, −15 dB, and −10 dB are presented via the delay value. In Figure 14b, it can be observed that the PDAR exhibits a certain degree of fluctuation as the delay value varies, which stems from the interaction between skywave signals and groundwave signals, specifically constructive interference or destructive interference of the two signals. When the delay value exceeds 300 μs, the interaction between the two signals weakens, and the PDAR tends to a constant value. In this case, skywave contributes to improved demodulation performance.

2. SIR

**Figure 15:**
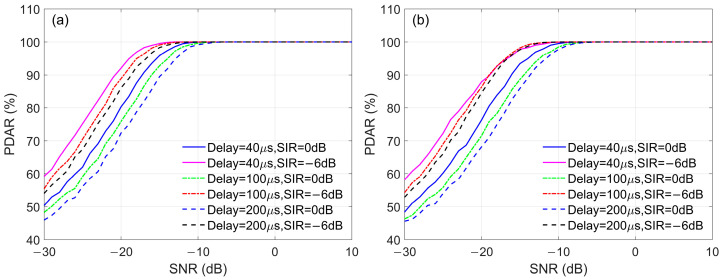
The impact of SIR on PDAR: (**a**) Reference has no noise; (**b**) SNR of reference pulse is −10 dB.

Figure 15 shows the impact of SIR on demodulation performance, where skywave delays are 40 μs, 100 μs, and 200 μs, respectively, and SIR is set to 0 dB and −6 dB. In Figure 15a, the SNR of the reference pulse has no random noise. However, the SNR of the reference pulse is −10 dB in Figure 15b. In Figure 15, it can be observed that when the SIR value decreases, the corresponding PDAR value increases under constant SNR of pulse to be demodulated, indicating better demodulation performance.

To provide a detailed quantitative reference for the simulation results presented above, Table 3 summarizes the specific PDAR values under various combinations of reference signal noise, skywave delays, and SIR levels. These data further corroborate the trends observed in Figure 14 and Figure 15, quantifying the impact of skywave interference intensity and delay on the final demodulation accuracy.

#### 3.2.3. CWI In-Band

**Figure 16:**
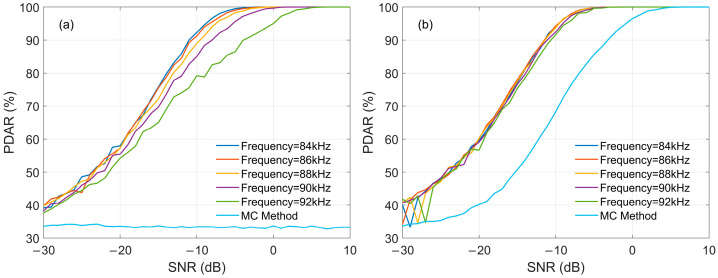
The impact of Integer-Period CWI on PDAR: (**a**) SIR = −6; (**b**) SIR = 12 dB.

CWI in-band is also a key factor affecting the demodulation performance of eLoran, and it mainly depends on the frequency and amplitude of the interference signal. If interference signal has integer-period during the single eLoran pulse duration, the SIR will be a key factor to be considered, which determines the judgment of whether a signal is interfered or not. Simulations are conducted following the processing flow shown in Figure 6, with the number of superimposed pulses set to 100. The SNR of the reference pulse varies with the SNR of the input signal. Figure 16 presents the impact of SIR on demodulation performance using MC-NF. In Figure 16a, where SIR = −6 dB, it indicates that the amplitude of the CWI is relatively large. The scenario of CWI interference can be determined via FFT, and the interference frequencies of the signal are 84 kHz, 86 kH, 88 kHz, 90 kH, and 92 kHz, respectively, with the calculation method being MC-NF. For comparison, the results using the MC method under different interference frequencies are also calculated, showing the average values under the above-mentioned interference frequency. It is obvious that MC-NF can effectively improve the demodulation performance. This is because the notch filter more strongly attenuates interference signals that are further away from the 100 kHz center frequency. Therefore, under a constant SNR, the larger the deviation between the interference frequency and the eLoran center frequency, the better the interference suppression effect and demodulation performance will be. Figure 16b, where SIR = 12 dB, represents a scenario where no CWI is determined, and the PDAR values are approximately equal under different interference frequencies.

**Figure 17:**
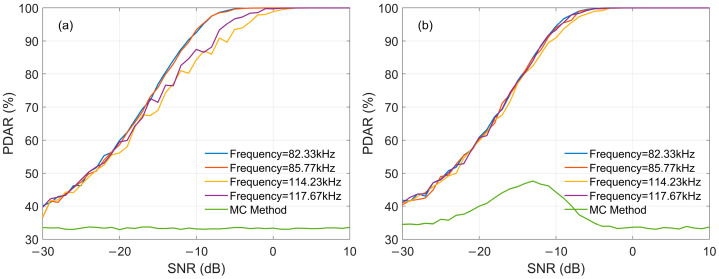
The impact of Non-Integer-Period CWI on PDAR: (**a**) SIR = −6; (**b**) SIR = 12 dB.

Similarly, if the interference signal has no integer-period during the single eLoran pulse duration, the SIR and interference phase will be considered. Figure 17a and Figure 17b present the results when the SIR is −6 dB and 12 dB, respectively. Figure 17a shows that there is CWI, and Figure 17b shows that there is no CWI. Figure 17 also verifies the effectiveness of the MC-NF method. It is important to note that the PDAR curves for the MC method in Figure 16 and Figure 17 represent the average performance across multiple interference frequencies.

Specifically, in Figure 17b, the MC method exhibits a fluctuation, where the PDAR slightly decreases at high SNRs. This phenomenon is attributed to the random initial phases of the non-integer CWI signals. Unlike the MC-NF method, which effectively filters out interference, the traditional MC method retains the interference components. Even under high SIR conditions, the superposition of the residual CWI with the eLoran signal is sensitive to phase randomness. Consequently, the correlation peak is unstable, leading to the observed performance fluctuation. This contrast further highlights the stability and robustness of the proposed MC-NF method, which maintains consistent performance by eliminating the interference source.

### 3.3. Performance Evaluation of GDAR

In Section 3.2, the differences in PDAR between the MC-NF and MC methods are compared in detail, especially the improvement in demodulation performance using the MC-NF method. In this section, the MC-NF combination method is applied to the pattern library, and we further analyze its demodulation performance, named PMC-NF. The performance evaluation of the PMC-NF algorithm is GDAR. As a reference, the calculation results of the single-pulse MC-NF, expressed in the form of GDAR, have also been computed. Here, we focus on describing the improvement in the PMC-NF method compared with MC-NF in demodulation performance, without specifically analyzing the reasons for it.

#### 3.3.1. Radom Noise and Skywave

**Figure 18:**
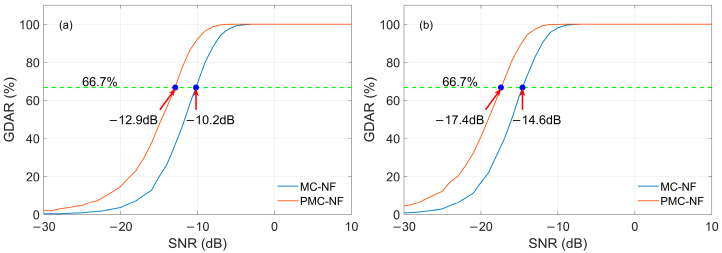
GDAR curves of different demodulation methods with random noise and skywave: (**a**) only random noise; (**b**) random noise and skywave.

The influence of noise and skywave using the MC-NF and PMC-NF methods are discussed in an environment without other interference. Figure 18 shows the GDAR curves of MC-NF and PMC-NF, where the SNR of the reference signal varies with the SNR of the input signal. Figure 18a shows the no skywave case, and Figure 18b includes the skywave, with a SIR of 0 dB and a skywave delay of 100 μs. It can be seen in Figure 18 that the PMC-NF method is superior to the MC-NF method, and it is even more superior to the MC method. In Figure 18a, when the GDAR value calculated by the PMC-NF method is 66.7%, the SNR of the received signal is −12.9 dB, while when the GDAR value calculated by the MC-NF is 66.7%, the SNR of the received signal is −10.2 dB. Similarly, in Figure 18b, the SNRs of the received signal corresponding to the three methods are −17.4 dB, −14.6 dB respectively. That is to say, compared with the MC-NF method, the PMC-NF method achieves a SNR improvement of approximately 2.8 dB.

#### 3.3.2. CWI In-Band

**Figure 19:**
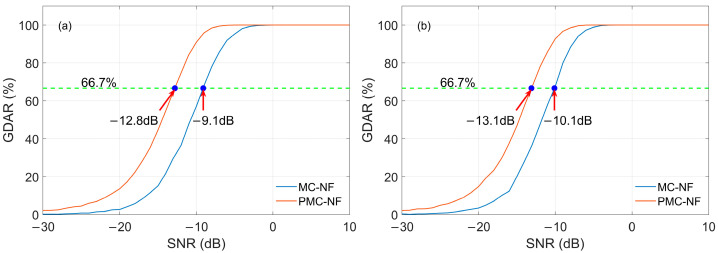
GDAR curves of different demodulation methods with CWI: (**a**) Interference frequency is 85 kHz; SIR = −6 dB; (**b**) Interference frequency is 82.33 kHz; SIR = −6 dB.

The influence of CWI in-band on demodulation using the MC-NF and PMC-NF methods is discussed. Figure 19 presents the GDAR curves of the two methods, where the interference frequencies in Figure 19a and Figure 19b are 85 kHz and 82.33 kHz, respectively. The SIR in these two cases is −6 dB, indicating that the amplitude of the CWI is relatively larger. In our simulation calculations, when the interference frequency is 85 kHz, the SNRs of the received signal corresponding to a GDAR value of 66.7% are −9.1 dB and −12.8 dB, corresponding to the MC-NF and PMC-NF methods. Similarly, when the interference frequency is 82.33 kHz, the SNRs are −10.1 dB and −13.1 dB. The results show that in the presence of CWI, the PMC-NF method can improve demodulation performance.

### 3.4. Overall Evaluation and Discussion

This section details the experimental design, result analysis, and comparative performance evaluation of the eLoran demodulation algorithms presented in this work, namely MC, MC-NF, and PMC-NF. Based on this investigation, the following conclusions are drawn:

1. The experimental results clearly show that the MC-NF method can effectively improve the demodulation performance of the enhanced Loran system compared to the MC method characterizing PDAR, including random noise, skywave, and CWI interference scenarios. These stem from the Notch Filtering’s suppression of noise and interference.
2. In most cases, the skywave signal is beneficial to the demodulation of the eLoran system. When the delay value is greater than 75 μs, the result is better than that without the skywave case. Meanwhile, the smaller the SIR, the higher the demodulation performance.
3. CWI in-band is a key factor affecting the demodulation performance of the eLoran system. It can also be suppressed by the MC-NF method, where the interference frequency is determined through frequency-domain analysis of the reference pulse.
4. The PMC-NF algorithm is a fusion of the MC-NF algorithm and the modulation pattern of eLoran, which further improves the demodulation performance of the enhanced Loran system characterizing GDAR. The SNR improvement of GDAR at 66.7% compared to MC-NF is approximately 2.8 dB.

In summary, this study validates the MC-NF algorithm as a robust and efficient method, demonstrating that it is highly capable for eLoran demodulation applications. At the same time, PMC-NF is a modification of MC-NF, which incorporates the pattern modulation. Comparative analysis shows that PMC-NF achieves higher demodulation performance than MC-NF; however, its computational complexity is also higher.

## 4. Discussion

### 4.1. Comparison with State-of-the-Art Methods

To further evaluate the performance and advantages of the proposed MC-NF and PMC-NF algorithms, we conducted a comparative analysis with four representative demodulation methods proposed in recent years: EC-PD (Yuan et al., 2020) [26], LLR (Lyu et al., 2022) [27], MSVM (Liu et al., 2024) [28], and PSSD (Liu et al., 2024) [29]. Table 4 summarizes the qualitative comparison regarding core techniques, robustness, complexity, reference signal conditions, and processing granularity.

It should be noted that the qualitative comparisons in Table 4, particularly regarding computational complexity and robustness, are derived from a theoretical analysis of the algorithmic principles and mathematical structures of each method.

Regarding complexity, the EC-PD method represents the baseline with low complexity due to its reliance on simple time-domain correlations (*O*(*N*)). In contrast, the LLR and MSVM methods involve complex probability calculations (exponential operations) and matrix–vector multiplications for kernel mapping, respectively, leading to higher computational costs. The PSSD and the proposed PMC-NF methods fall into the medium range, as they incorporate FFT-based spectral processing (*O*(*N*\log*N*)), which is slightly more intensive than simple correlation but significantly lighter than iterative optimization or deep learning inference.

Regarding robustness, the ‘High’ or ‘Very High’ robustness of PMC-NF is attributed to its deterministic interference suppression mechanism. While statistical methods (like LLR) often assume Gaussian noise models and degrade under non-Gaussian CWI, and time-domain correlation (like EC-PD) lacks frequency selectivity, the proposed method explicitly identifies and nullifies the CWI energy via notch filtering. This structural advantage ensures superior stability in high-interference scenarios without requiring extensive training data like MSVM.

### 4.2. Analysis of Performance and Practicality

The comparison highlights three critical distinctions that demonstrate the system-aware nature and engineering practicality of the proposed PMC-NF method:

1. System-Aware Pattern Processing vs. Single-Pulse Processing: Most existing methods, including EC-PD and recent machine learning methods, focus primarily on PDAR, treating each pulse as an independent classification task. In contrast, the proposed PMC-NF is designed with the specific encoding structure of the eLoran system in mind. By utilizing pattern matched correlation, it leverages the inter-pulse correlation within a pulse group. This allows the system to correct errors in individual pulses using the structural information of the group, significantly improving GDAR. As shown in our results, even when single-pulse conditions degrade, the pattern-level processing maintains high link reliability.
2. Robustness with Realistic Noisy References: It is crucial to note that the performance gains of PMC-NF were achieved under rigorous conditions. While methods like EC-PD and machine learning methods often utilize ideal or perfectly synchronized reference signals in simulations, our experiments utilized a noisy reference signal reconstructed via LDA from the received stream. This setup mimics the actual “blind” operating environment of a receiver. Despite the noise residue in the reference signal (which typically degrades correlation gain), the PMC-NF method achieved superior demodulation rates, proving its robustness in practical scenarios where a clean reference is unavailable.
3. Complexity and Feasibility: While machine learning methods demonstrate impressive theoretical performance, their high computational complexity and demand for extensive training data pose significant challenges for deployment on low-cost, low-power embedded receivers (e.g., FPGA or MCU). The EC-PD method is computationally efficient but struggles with strong in-band Continuous Wave Interference (CWI). The proposed PMC-NF integrates the spectral efficiency of notch filtering with the coding gain of pattern matching. It maintains a low computational footprint, similar to standard correlation methods, while offering interference immunity comparable to complex learning models, making it highly suitable for real-time hardware implementation.

### 4.3. Computational Complexity Analysis

To assess the real-time feasibility of the proposed algorithms, we analyzed the computational costs of MC, MC-NF, and PMC-NF. The computational complexity is primarily determined by the correlation length (*N*), the Fast Fourier Transform (FFT) for interference detection, and the filtering operations.

The traditional MC method is the most computationally efficient, requiring only *N* multiply–accumulate (MAC) operations for the correlation process. Its complexity is *O*(*N*).

The MC-NF method introduces two additional stages: interference detection and notch filtering. The detection stage typically utilizes an *N*-point FFT, with a complexity of *O*(*N*log*N*). However, in practical engineering, detection does not need to be performed for every pulse; it can be executed periodically. The IIR notch filter adds a small constant number of operations per sample, typically ranging from 5 to 10 MACs for a second-order filter, thereby maintaining an overall linear complexity *O*(*N*) for the filtering stream.

The PMC-NF method shares the same filtering and detection architecture as MC-NF. The primary difference lies in the pattern matching, which involves a longer correlation sequence (Pulse Group length). However, since the underlying operation remains a linear correlation, the complexity growth is linear.

To strictly evaluate the engineering feasibility, we summarize the computational costs of the proposed methods in Table 5. We define the complexity of the MC method as the baseline.

While MC-NF and PMC-NF entail a moderate increase in computational load compared to MC, primarily stemming from the FFT module and IIR filters, this cost is significantly lower than that of complex matrix inversion methods or machine learning models, which require massive parallel computations. Therefore, the proposed methods strike a favorable balance between performance enhancement and computational efficiency.

### 4.4. Hardware Implementation and Real-World Considerations

Although this study relies on extensive simulations, the experimental design explicitly considered factors limiting real-world performance, such as dynamic noise and multipath effects (skywave). The use of a noisy reference signal reconstructed via LDA closely mimics the “blind” operation of a physical receiver.

For hardware implementation, the proposed PMC-NF algorithm is highly suitable for Field-Programmable Gate Array (FPGA) deployment. The core components of IIR notch filters and correlators utilize standard DSP slices in the form of multipliers and adders, which are abundant in modern FPGAs. Unlike floating-point intensive algorithms, the proposed structure can be easily adapted to fixed-point arithmetic with minimal precision loss.

However, we acknowledge that real-world measurements may introduce additional challenges, such as non-Gaussian impulsive noise and rapid phase variations caused by receiver motion. It is expected that the demodulation performance in field tests may show slight degradation compared to simulation results. Future hardware-in-the-loop (HIL) tests will be crucial to optimize the notch filter’s response time and the pattern matching synchronization threshold.

## 5. Conclusions

With the rapid development of ground-based navigation systems, the eLoran system has become a research focus. Demodulation and decoding are a key link affecting the reception performance of the eLoran system.

This study introduces the principles, influencing factors, and limitations of traditional MC algorithms. By combining the notch filter with the MC method, a demodulation processing method of MC-NF is proposed firstly. This method suppresses the impact of random noise through notch filtering of the fixed-frequency eLoran signal; meanwhile, the judgment and notch processing of in-band CWI can reduce the influence of interference. Experimental results clearly show that the proposed MC-NF method can effectively improve the demodulation performance of the eLoran system compared to the MC method characterizing PDAR, further improving the decoder effectively. Secondly, by combining the pattern modulation method of eLoran with the MC-NF method, a new PMC-NF method is proposed. Essentially, the MC-NF method and PMC-NF method are consistent in principle, but PMC-NF increases the data length, which can further improve the demodulation performance. The results show that the PMC-NF method can improve the results by approximately 2.8 dB compared to MC-NF when charactering GDAR.

Although extensive simulation experiments have verified the effectiveness of the proposed MC-NF and PMC-NF methods, particularly in complex environments, this study currently lacks validation via field measurement data. Consequently, verifying these application effects in a physical eLoran receiver remains a critical next step. Future work will focus on three main areas: (1) Optimizing the judgment criterion for in-band CWI and extending the algorithm to address multi-frequency interference scenarios; (2) Implementing the PMC-NF algorithm on an FPGA-based software-defined radio (SDR) platform to evaluate its real-time resource consumption and latency; and (3) Conducting field trials in complex electromagnetic environments to verify the algorithm’s robustness against real-world impulsive noise and dynamic interference. These efforts will bridge the gap between theoretical simulation and practical engineering application.

## Figures and Tables

**Table 1:** Corresponding relationship between patterns and ASCII codes (partial).

ASCII	Binary Data	Pattern	ASCII	Binary Data	Pattern
0	0000000	00−−++	...	...	...
1	0000001	00−+−+	123	1111011	++−−00
2	0000010	00−++−	124	1111100	000+0−
3	0000011	00+−−+	125	1111101	00+−00
4	0000100	00+−+−	126	1111110	00+0−0
...	...	...	127	1111111	00+00−

**Table 2:** The results of the matched correlations for different samplings.

Sampling (MHz)	$f_{s}\cdot E_{kk}$	${f_{s}\cdot E}_{km}$	$f_{s}\cdot \left(E_{kk}-E_{km}\right)$
1	41.59	33.64	7.95
2	83.18	67.28	15.90
3	124.76	100.92	23.84
4	166.35	134.56	31.79
5	207.94	168.20	39.74
6	249.53	201.84	47.68
7	291.12	235.48	55.63
8	332.71	269.12	63.58
9	374.29	302.76	71.53
10	415.88	336.40	79.88

**Table 3:** The result of the PDAR effected by skywave interference with SIR.

Reference Signal (dB)	Delay (μs)	SNR of Signal (dB)	PDAR (%)	
			SIR = 0 dB	SIR = −6 dB
No noise	40	−30	50.37	59.23
		−20	79.81	92.08
		−10	99.92	100.00
	100	−30	48.31	55.43
		−20	75.61	88.89
		−10	100.00	100.00
	200	−30	45.96	54.05
		−20	72.36	86.08
		−10	99.18	100.00
−10 dB	40	−30	48.36	58.16
		−20	76.30	87.88
		−10	99.54	99.98
	100	−30	46.31	54.28
		−20	71.48	86.27
		−10	98.48	98.98
	200	−30	45.60	52.87
		−20	68.16	84.56
		−10	97.73	98.98

**Table 4:** Comparison of recent eLoran demodulation methods.

Method	Core Technique	Reference Signal	Processing Granularity	Computational Complexity (Relative to MC)	Robustness to CWI (Min. Tolerable SIR)
EC-PD	Envelope Correlation + Phase Detection	Ideal (Simulation)	Single Pulse (PDAR focus)	Low (≈1.0×)	Low (>0dB)
LLR	Log-Likelihood Ratio	Ideal	Single Pulse/Bit	Medium (≈1.5×)	Medium (>−5dB)
MSVM	Multiclass SVM (Machine Learning)	Ideal (Implicitly learned)	Single Pulse (PDAR focus)	High (≈4.5×)	High (≈−10dB)
PSSD	Pulse Stacking + Spectral Density	Ideal (Requires precise stacking)	Single Pulse (PDAR focus)	Medium (≈1.35×)	High (≈−10dB)
MC-NF	Matched Correlation + Notch Filter	Noisy (Recovered via LDA)	Single Pulse (PDAR and GDAR)	Low (≈1.15×)	High (<−10dB)
PMC-NF	Pattern Matching + Notch Filter	Noisy (Recovered via LDA)	Pulse Group (GDAR)	Medium (≈1.35×)	Very High (<−13dB)

Note: (1) Computational Complexity: Quantified as the relative execution time normalized to the standard MC method. “Low” indicates <1.2×, “Medium” indicates 1.2×~2.0×, and “High” indicates >4.0×. (2) Robustness to CWI: Quantified by the minimum tolerable SIR. “Low” indicates SIR > 0 dB, “Medium” indicates SIR > −5 dB, “High” indicates SIR ≈ −10 dB, and “Very High” indicates SIR < −12 dB.

**Table 5:** Comparison of computational complexity and costs for the proposed methods.

Method	Main Operations	Computational Complexity Order	Relative Calculation Cost
MC	Single Pulse Correlation	*O*(*N*)	1.00×
MC-NF	FFT + Notch Filtering + Pulse Correlation	*O*(*N*) (Amortized)	≈1.15×
PMC-NF	FFT + Notch Filtering + Pattern Matching	*O*(*M*·*N*)	≈1.35×

Note: The Relative Calculation Cost is estimated based on the number of floating-point operations required per second, normalized to the MC method.

## Data Availability

The data supporting the results presented in this paper are not completely publicly available at this time but may be obtained from the authors upon reasonable request.

## Abbreviations

MC	Matched Correlation
MC-NF	Matched Correlation Demodulation Algorithm Integrates Notch Filtering
PMC-NF	Pattern Modulation-Matched Correlation Demodulation Algorithm Integrates Notch Filtering
PPM	Pulse Position Modulation
CWI	Continuous Wave Interference
PDAR	Pulse Demodulation Accuracy Rate
GDAR	Group Demodulation Accuracy Rate
FDAR	Frame Demodulation Accuracy Rate
LDA	Linear Digital Averaging

## References

[1] Dardanelli G., Maltese A. (2022). On the accuracy of cadastral marks: Statistical analyses to assess the congruence among GNSS-based positioning and official maps. Remote Sens..

[2] Lee H., Seo J., Kassas Z.Z.M. (2022). Urban road safety prediction: A satellite navigation perspective. IEEE Intell. Transp. Syst. Mag..

[3] Lee Y., Hwang Y., Ahn J.Y., Seo J., Park B. (2023). Seamless Accurate Positioning in Deep Urban Area Based on Mode Switching Between DGNSS and Multipath Mitigation Positioning. IEEE Trans. Intell. Transp. Syst..

[4] Specht C. (2023). Maritime DGPS System Positioning Accuracy as a Function of the HDOP in the Context of Hydrographic Survey Performance. Remote Sens..

[5] Lu T., Sun Y., Zhu Q., Zhou X., Li Q., Liu J. (2025). Intelligent Upgrading of the Localized GNSS Monitoring System: Profound Integration of Blockchain and AI. Electronics.

[6] Liu K.Q., Yuan J.B., Yan W.H., Yang C.Z., Guo W., Li S.F., Hua Y. (2022). A Shrink-Branch-Bound Algorithm for eLoran Pseudorange Positioning Initialization. Remote Sens..

[7] Son P.W., Park S.G., Han Y., Seo K., Fang T.H. (2023). Demonstration of the Feasibility of the Korean eLoran System as a Resilient PNT in a Testbed. Remote Sens..

[8] Dinesh S. (2013). Global Navigation Satellite System (GNSS) Spoofing: A Review of Growing Risks and Mitigation Steps. Def. ST Tech. Bull..

[9] Grant A., Williams P., Ward N., Basker S. (2009). GPS Jamming and the Impact on Maritime Navigation. J. Navig..

[10] Sukhenko A., Meirambekuly N., Syzdykov A., Mukhamedgali A., Mellatova Y. (2025). GNSS for High-Precision and Reliable Positioning: A Review of Correction Techniques and System Architectures. Appl. Sci..

[11] Hussain A., Akhtar F., Khand Z.H., Rajput A., Shaukat Z. (2021). Complexity and Limitations of GNSS Signal Reception in Highly Obstructed Environments. Eng. Technol. Appl. Sci. Res..

[12] Zhao X., Zhan X.Q., Liu X., Li S.J. (2014). GNSS Vulnerability Analysis and Assessment. J. Aeronaut. Astronaut. Aviat..

[13] Johnson G., Shalaev R., Hartnett R., Swaszek P., Narins M. (2005). Can LORAN meet GPS backup requirements?. IEEE Aerosp. Electron. Syst. Mag..

[14] Yang M., Yan B., Yang C., Jiang X., Li S. (2025). Research on the eLoran/GNSS Combined Positioning Algorithm and Altitude Optimization. Remote Sens..

[15] Ward N., Hargreaves C., Williams P., Bransby M. Delivering resilient PNT. Proceedings of the 2015 International Association of Institutes of Navigation World Congress (IAIN).

[16] Williams P., Basker S., Ward N. (2008). e-Navigation and the Case for eLoran. J. Navig..

[17] Liu S.Y., Hua Y., Zhang S.G. (2023). Weight-Function Notch Filter Algorithm for Narrow-band Interference in eLORAN System. J. Electron. Inf. Technol..

[18] Zhang K., Yang F., Wang W., Ye X. (2024). eloran signal message prediction algorithm based on Alexnet-ECA. IET Radar Sonar Navig..

[19] Johnson G.W., Swaszek P.F., Hartnett R.J., Shalaev R., Wiggins M. An Evaluation of eLoran as a Backup to GPS. Proceedings of the 2007 IEEE Conference on Technologies for Homeland Security.

[20] Liu S.Y., Zhang S.G., Hua Y. (2022). A Cycle Identification Algorithm for enhanced LOng RAnge Navigation Signal Based on Skywave Reconstruction Technology. J. Electron. Inf..

[21] Yan W.H., Zhao K.J., Li S.F., Wang X.H., Hua Y. (2020). Precise Loran-C Signal Acquisition Based on Envelope Delay Correlation Method. Sensors.

[22] Zhang K., Yang F., Wang W., Wang B. (2025). ELoran Signal Message Recognition Algorithm Based on GTCN-Transformer. IET Radar Sonar Navig..

[23] Li J.Y. (2006). Design and Implementation of Loran-C Datalink. Inf. Elect. Eng..

[24] Li S.F., Wang Y.L., Hua Y., Xu Y.L. (2012). Research of Loran-C data demodulation and decoding technology. Chin. J. Sci. Instrum..

[25] Lo S.C., Peterson B.B., Enge P.K. (2007). Loran Data Modulation: A Primer [AESS Tutorial IV]. IEEE Aerosp. Electron. Syst. Mag..

[26] Yuan J.B., Yan W.H., Li S.F., Hua Y. (2020). Demodulation Method for Loran-C at Low SNR Based on Envelope Correlation–Phase Detection. Sensors.

[27] Lyu B.Y., Hua Y., Yan W.H., Yuan J.B., Li S.F. Data demodulation algorithm of enhanced Loran system. Proceedings of the International Conference on Electronic Information Technology (EIT 2022).

[28] Liu S.Y., Yan B.R., Guo W., Hua Y., Zhang S.G., Lu J., Xu L., Yang D. (2024). Research on ELoran Demodulation Algorithm Based on Multiclass Support Vector Machine. Remote Sens..

[29] Liu S.Y., Yan B.R., Hua Y., Kou W.D., Zhang S.G., Xu L., Lu J. (2024). Phase Spectrum Smoothing Demodulation: A New Frontier in eLoran Signal Processing for Enhanced Performance. Remote Sens..

[30] Williams P., Last D. Modelling Loran-C envelope-to-cycle differences in mountainous terrain. Proceedings of the 32nd Annual Meeting, International Loran Association.

[31] Yan W.H., Dong M., Li S.F., Yang C.Z., Yuan J.B., Hu Z.P., Hua Y. (2022). An eLoran Signal Cycle Identification Method Based on Joint Time–Frequency Domain. Remote Sens..

[32] Liu S.Y., Zhang S.G., Hua Y. (2022). Research on GRI Combination Design of eLORAN System. J. Electron. Inf..

[33] Safar J., Williams P., Grant A., Vejrazka F. (2016). Analysis, modelling and mitigation of cross-rate interference in eLoran. J. Navig..

[34] Offermans G.W.A., Helwig A.W.S., Willigen D.V. (1999). Eurofix system and its developments. J. Navig..

[35] Van Willigen D., Offermans G.W.A., Helwig A.W.S. EUROFIX: Definition and current status. Proceedings of the IEEE 1998 Position Location and Navigation Symposium.

[36] (2018). Transmitted Enhanced Loran (eLoran) Signal Standard for Tri-State Pulse Position Modulation.

[37] Wu H.T., Li X.H., Zhang H.J., Gao H.J., Bian Y.J. UTC message broadcasting over Loran-C data channel. Proceedings of the 2002 IEEE International Frequency Control Symposium and PDA Exhibition (Cat. No.02CH37234).

[38] Lo S.C., Peterson B.B., Enge P.K., Swaszek P. (2007). Loran data modulation: Extensions and examples. IEEE Trans. Aerosp. Electron. Syst..

[39] Helwig A., Offermans G., Stout C., Schue C. (2011). eLoran System Definition and Signal Specification Tutorial.

[40] Jia R., Li Y., Qu D. (2025). Improving Data Communication of Enhanced Loran Systems Using 128-ary Polar Codes. Sensors.

[41] Ahmad M., Rahman S. (2014). Design of HDLC Controller with CRC Generation Using VHD. Int. J. Mod. Eng. Res..

[42] Wu B., Li Y., Zhang D.L. (2011). Based on reed solomon code design of a flash memory controller. Electr. Measur. Technol..

[43] Wu M., Jin X., Qi X., Di J., Yu T., Li F. (2025). An Zero-Point Drift Suppression Method for eLoran Signal Based on a Segmented Inaction Algorithm. Electronics.

[44] Su J.F., Wu H.T., Wang Y.L., Bain Y. (2006). The Study of Loran-C Datalink Application in BPL Time Service. Acta Astron. Sin..

[45] (1991). Generic Specification for Marine Loran-C Receiving Equipment. https://openstd.samr.gov.cn/bzgk/std/newGbInfo?hcno=96678FAC72C6858C7E7110B12BE7EA2B.

[46] Kim S.Y., Kang C.H., Park C.G. (2019). Frequency tracking and mitigation method based on CPHD filter and adaptive multiple linear Kalman notch filter for multiple GNSS interference. Navigation.

